# High-density biomass as a substrate for stimulating biosulfidogenesis in the deep layer of stratified acidic pit lakes

**DOI:** 10.1128/aem.02369-25

**Published:** 2026-01-12

**Authors:** Yutong Liu, Rachel A. Brennan, Javier Sánchez-España, Carlos Vilchez, Juan-Luis Fuentes, Jennifer L. Macalady, William D. Burgos

**Affiliations:** 1 Department of Civil and Environmental Engineering, The Pennsylvania State University 8082, University Park, Pennsylvania, USA; 2 Department of Civil and Environmental Engineering, Colorado State University 3447, Fort Collins, Colorado, USA; 3 Department of Planetology and Habitability, Centro de Astrobiología (CAB), Spanish National Research Council (CSIC) 120422, Torrejón de Ardoz, Madrid, Spain; 4 Algal Biotechnology Group, CIDERTA and Faculty of Experimental Sciences, University of Huelva 16743, Huelva, Spain; 5 Research Center of Natural Resources, Health and the Environment (RENSMA), University of Huelva 16743, Huelva, Spain; 6 Department of Geosciences, The Pennsylvania State University 8082, University Park, Pennsylvania, USA; Colorado School of Mines, Golden, Colorado, USA

**Keywords:** bioremediation, acidic pit lake, high-density biomass, metal(loid) removal, sulfate reducing bacteria

## Abstract

Accumulation of harmful metal(loid)s in acidic pit lakes (APLs) is a serious environmental issue in mining districts. This lab-based study evaluated a novel method to stimulate dissimilatory sulfate reduction to promote the formation of sparingly soluble metal(loid)-sulfide minerals in the permanently stratified deep layer of Cueva de la Mora (CM), an APL in the Iberian Pyrite Belt in Spain. Solid-phase biomass was selected because it can be pressed into high-density forms that are dense enough to settle into the deep layer of a lake. This “direct delivery” of electron donor overcomes the current “indirect method” to stimulate algae growth in the upper layer and wait for algae to die and settle into the deep layer. We added the microalgae *Coccomyxa onubensis* (predominant in CM), *Euglena gracilis* (another acid-tolerant microalgae), and *Lemna obscura* (duckweed), as well as model biocomponents (amino acids, monosaccharides, and lipids) as substrates to stimulate biological sulfide production (biosulfidogenesis). We found that compared with biocomponents, high-density biomass required a shorter lag time before it was utilized. Temporal patterns of the production of sulfide and volatile fatty acids with high-density biomass were similar to patterns with amino acids, suggesting that amino acids may be the preferred substrate among the biocomponent monomers for the microbial community. Biosulfidogenesis led to the complete removal of metal(loid)s (Zn and As) contaminants from solution, mimicking the chemical composition of the deep layer. *Desulfosporosinus*, the only acid-tolerant sulfate-reducing bacteria (SRB) identified *in situ*, was significantly enriched in the laboratory setup and presumably responsible for biosulfidogenesis.

**IMPORTANCE:**

Remediation of high concentrations of harmful metal(loid)s in acidic pit lakes is challenging. This research presents a novel strategy by supplying high-density biomass as a carbon source and electron donor to stimulate biological dissimilatory sulfate reduction in acidic pit lakes in the Iberian Pyrite Belt. The formation of biogenic sulfide precipitates dissolved metal(loid)s in the acidic pit lakes. This approach is feasible in meromictic acidic pit lakes, where precipitated metal(loid)s would remain sequestered in bottom sediments. However, the deep layer of acidic pit lakes is often oligotrophic with respect to organic carbon. Pelletized high-density biomass can be added to the top layer of the lake and transported to the deep layer. This strategy offers practical and adaptable guidance for the bioremediation of persistent metal(loid) contamination in acidic pit lakes.

## INTRODUCTION

Acidic pit lakes (APLs) form when open cast mines are abandoned and allowed to flood. Exposure of polymetallic sulfide ores and adjacent mine spoils leads to the formation of acid mine drainage (AMD) in the lakes. Abandonment of open cast mines creates significant environmental problems due to low pH and high concentrations of metal(loid) in the lake water (1).

A conventional approach for the remediation of APLs is by chemical neutralization. This process involves the addition of alkalinity in the form of lime (CaO), hydrated lime (Ca(OH)_2_), soda ash (Na_2_CO_3_), or caustic soda (NaOH). Chemicals can be added to a lake as dry solids, wet slurries, or concentrated liquid solutions. Neutralization aims to treat the whole lake volume and eventually blend layers that might otherwise remain permanently separate due to stratification. This approach may also employ pumps for active circulation of the lake contents and treatment of the recirculated water before its return to the lake. This method is often cost-prohibitive (2).

An unconventional approach for the remediation of a meromictic (permanently stratified) APL would be to first treat only the deep layer of the lake. Concentrations of metal(loid) contaminants are typically considerably higher in the deep layer compared with the upper layer or chemocline. The transport of high concentrations of toxic metal(loid)s from the deep layer into adjacent (ground)water systems could be the greatest risk from an APL.

Biologically mediated neutralization of APLs has proven challenging. Although sulfate is abundant in the deep layer, the availability of suitable organic substrates is often a limiting factor for dissimilatory sulfate reduction; therefore, the addition of organic substrates is commonly recommended in bioremediation practices (3–7). In addition, possible biological sulfide oxidation (via a cryptic sulfur cycle) may also limit sulfide accumulation (8, 9). Furthermore, there have been only a few acid-tolerant sulfate-reducing microorganisms identified or studied, making the scarcity of suitable microorganisms another limiting factor. A final problem is related to the re-oxygenation of the deep layer and reoxidation of sulfides when holomictic pit lakes mix and overturn in the winter season (1). Therefore, biologically mediated neutralization is feasible only in meromictic pit lakes (10).

Delivering an abundant amount of organic carbon into the deep layer of an APL should stimulate sulfate reduction and minimize sulfide reoxidation (11). Biogenic sulfide would then readily react with soluble metal(loid)s to form sparingly soluble metal(loid)-sulfide precipitates and accumulate in the lake bottom sediments. Because permanent stratification of an APL is due to differences in water density between the lake layers, substrate addition would have to be carefully monitored to avoid removing excessive dissolved species from the deep layer.

Nutrient fertilization to grow algae in the upper layer of APLs was previously attempted in Lusatia mining districts, Germany. The lakes formed from lignite mining were selected because blooms of green algae had been observed even with low pH (≤ 3) and high iron concentrations (12). Nutrient addition to stimulate more algae growth promoted sulfate reduction, generated alkalinity, and removed metal(loid)s from the lake (13). This process, however, proved partially unsuccessful because the lake selected for the demonstration project was shallow and prone to seasonal, temperature-driven turnover, so that sulfide was re-oxidized. The amount of alkalinity generated from sulfate reduction was also shown to be relatively low compared with the acidity in the lake, thus requiring repeated fertilization events and considerable time (14–16). Instead, many of the German APLs were remediated via chemical neutralization (17, 18). Another example is the Berkeley Pit Lake in Montana, USA, a holomictic system that experiences seasonal turnover. During these mixing events, oxidants are redistributed throughout the water column, resulting in the reoxidation of sulfide precipitates (19).

Cueva de la Mora (CM), located in the Iberian Pyrite Belt (IPB) in SW Spain, is one of the most studied APLs in the world. CM exhibits typical features of these lakes, including low pH, high sulfate concentrations, and an abundance of metal(loid)s, particularly in its deep layer (9, 20). The total organic carbon (TOC) concentration in the deep layer of CM (~3 mg/L) is only slightly lower than the global lake average (~5.6 mg/L) (21, 22). Nevertheless, previous studies have indicated that organic carbon remains a limiting factor for dissimilatory sulfate reduction in the deep layer of CM and that supplementing organic substrates is a feasible strategy to stimulate this process (9, 11). Bioremediation of the deep layer of CM can mitigate risks to human and environmental health posed by extremely high concentrations of metal(loid)s (23–25). Its physical-chemical status as a permanently stratified lake eliminates concerns from metal(loid) reoxidation and acidity regeneration due to seasonal water turnover.

Notably, the surface layer and chemocline of CM contain substantial amounts of microalgae. In both layers, 96%–98% of eukaryotes belong to the genus *Coccomyxa*. In contrast, no microalgae were detected in the deep layer, which is devoid of any solar light to allow photosynthesis (26–28). Sulfide accumulation has only been detected in the anoxic portion of the chemocline and not in the deep layer (29). This is an unusual pattern, as the deep layer is more reduced and would theoretically favor dissimilatory sulfate reduction (21). It is suspected that sulfide accumulation in the chemocline is due to an abundance of algae that serves as a substrate for dissimilatory sulfate reduction. Organic compounds released by the decomposition of settling phytoplanktonic biomass, however, were rapidly consumed by sulfate-reducing bacteria (SRB) in the chemocline and would not reach deeper levels (21).

High-density biomass refers to biomass that has been modified to be denser than CM lake water, enabling it to sink rapidly to the lake bottom in this study. This “direct delivery” of electron donor overcomes problems with the current “indirect method” to stimulate algae growth in the upper layer and wait for algae to die and settle into the deep layer. The objectives of this laboratory-based research were to (i) determine the potential effectiveness of several types of high-density biomass to stimulate dissimilatory sulfate reduction, (ii) identify what compositional components of the biomass promoted dissimilatory sulfate reduction by the microbial community, and (iii) evaluate how different types of organic substrates influence microbial community composition and their correlation with the rate and extent of dissimilatory sulfate reduction.

## MATERIALS AND METHODS

### Research site and sample collection

Cueva de la Mora (CM) is a ~40-meter-deep APL located in the northern part of the province of Huelva, Spain, in the Iberian Pyrite Belt. Mining activities at CM involved both underground and open-cast methods starting in 1875, with the site eventually being abandoned in the early 1970s (8, 29). CM is classified as a meromictic APL, with a surface layer extending to ~10 m in depth and a deep layer situated below the chemocline at ~12 m depth. The surface layer is oxygen-saturated, whereas the deep layer is anoxic (DO < 0.1 mg/L). The sulfate and ferrous ion concentrations are significantly higher in the deep layer (~12 g/L SO₄²⁻ and ~6 g/L Fe(II)) compared with the surface layer (~2 g/L SO₄²⁻ and ~0.1 g/L Fe(II)). Additionally, the deep layer exhibits a higher pH (4.2–4.6) compared with the surface layer (pH 2.7–3.1) (29).

Microbial biomass from the deep layer of CM was collected from a boat attached to a buoy located above the deepest part of the lake in October 2021. Sample collection, preservation, and transport were described previously (11). Briefly, an electric high-lift membrane pump was used to pump water from 34 m below the lake surface (below the chemocline) through a stainless-steel filter assembly. The filter assembly was 142 mm in diameter and held 0.22 μm PES filters. A total of ca. 10 L of water was pumped through each filter before filtrate production ceased. After filtration, each filter (20 total) was rolled and slid into a 30 mL sterile glass serum tube, filled with water from the deep layer, sealed with thick rubber stoppers and aluminum crimps, and purged with N_2_ gas for 5 min and then over-pressurized. Serum tubes were placed in a cooler on ice and transported back to Penn State University. Upon arrival, all tubes were stored at 4°C until further use.

### Medium preparation

Medium was designed to match the water chemistry of the deep layer of CM (20, 26, 28, 29). N and P were added based on cell synthesis requirements (using biomass formula of C_5_H_7_O_2_N_1_P_0.1_). Medium contained (per liter): 3,000 mg FeSO_4_·7H_2_O, 219 mg CaSO_4_·2H_2_O, 202 mg MgSO_4_, 69.5 mg NH_4_Cl, 48.0 mg ZnSO_4_·7H_2_O, 35.8 mg MnSO_4_·H_2_O, 34.0 mg Al_2_(SO_4_)_3_·H_2_O, 6.20 mg NaCl, 3.82 mg NaH_2_AsO_4_, 0.97 mg KCl, 0.82 mg CoCl_2_·6H_2_O, 0.57 mg NaHCO_3_, 0.43 mg NaNO_3_, 0.37 mg NiCl_2_·6H_2_O, 0.04 mg CuSO_4_, and 0.174 mL 1 N H_3_PO_4_. The medium was first prepared without the addition of FeSO_4_·7H_2_O and adjusted to pH 4.2. The medium was then passed through a 0.2 µm bottle-top filter to remove any undissolved solids. Subsequently, 100 mL of medium was dispensed into 165 mL serum bottles (DWK Life Sciences, Millville, NJ, USA) and then purged with N_2_ for 20 min using long needles submerged into the liquid. During purging, FeSO_4_·7H_2_O was added to the medium to avoid oxidation of Fe(II). After purging the liquid phase, the headspace was also purged for 20 min with N_2_. Serum bottles containing the medium were then autoclaved at 121°C, 21 psi, for 30 min. Once cooled, the bottles were moved into an anaerobic chamber. One milliliter of 100× Wolfe vitamin solution (30) was added to each bottle after inoculation.

### Selection of substrates

High-density biomass and individual biocomponents were evaluated as substrates to stimulate sulfate reduction. *Coccomyxa onubensis* (hereafter referred to as *Coccomyxa*), the predominant algae in both the surface layer and chemocline of CM, was provided by the Algal Biotechnology Group of the University of Huelva. *C. onubensis* was previously deposited at the Culture Collection of Goettingen University (SAG). *C. onubensis* was dried in an oven with fan-assisted circulation (JP Selecta DRY-BIG 2002972, Barcelona, Spain) and converted into a powder of grain size B100 mm using a vibratory disc mill (Retsch GmbH RS100, Haan, Germany) (31). The particle size of *Coccomyxa* powder was less than 3.35 mm, as determined by a sieve test (Fig. S1).

*Euglena gracilis* (hereafter referred to as *Euglena*), another microalgae common in acidic environments (32, 33), was purchased from ALFA Chemistry in New Jersey, USA, in the form of sterilized fine powder (particle size < 0.45 mm). Its yellow appearance results from carotenoid pigments within the cells (34, 35) (Fig. S1).

Duckweed was selected as another substrate because it can grow under a wider range of conditions (36), compared with acidophilic algae such as *Coccomyxa* and *Euglena*. Duckweed can grow over a range of pH values (3–10) and temperatures (15°C–35°C) (37). Duckweed generally grows rapidly with sufficient light in calm water and can be grown in municipal wastewater (38). Duckweed was provided by the Brennan research group at Penn State in the form of compressed pellets (39). The composition of duckweed varies significantly depending on its growth conditions. The duckweed used in this study was cultivated in an outdoor pond fed treated municipal wastewater with relatively low concentrations of nitrogen and phosphorus and had previously been identified as a monoculture of *Lemna obscura* (40). This duckweed had a lower protein content (~11.6%), compared with duckweed grown in a nutrient-rich environment (39, 41). The duckweed was pelletized using a Pellet Pros farm-scale, open hopper batch pelletizer (Model PP220, Dubuque, Iowa, USA), which produced ~2.5 cm long, 0.16 mm radius pellets. Duckweed pellets were ground into a powder (particle size < 0.18 mm) before use (Fig. S1).

High-density biomass samples were characterized by proximate and elemental analyses. Compositional analyses for each type of high-density biomass were obtained from the literature. Proximate analyses were conducted by Cumberland Valley Analytical Services (Waynesboro, PA, USA) to determine dry mass percentages of starch, protein, fat, and fiber using Official Methods of Analysis (42). Elemental analyses were conducted by the Penn State University Agricultural Analytical Services Lab to determine C, N, P, S, and metal concentrations using Test Methods for the Examination of Composting and Compost (TMECC) methods 4.02, 4.03, and 4.05 (43).

Three major classes of biocomponents—amino acids, monosaccharides, and lipids—were also chosen as substrates. Casamino acids are a blend of amino acids commonly used in studies of SRB metabolism (44). Glucose and galactose were selected to represent monosaccharides; both are extensively found in microalgal polysaccharides (45). Representative long-chain fatty acids (LCFAs), namely palmitate (C16:0) and oleate (C18:1), were selected to represent lipids based on their presence as primary lipid components in *Coccomyxa* (46, 47).

### Experimental setup

In previous experiments, 5 mM glycerol was added to stimulate sulfate reduction (11). This concentration, expressed as chemical oxygen demand (COD), was used to set the equivalent concentration for certain substrates tested in the current study. This concentration was measured as 494 mg O_2_/L using a HACH COD kit and is equivalent to 100% mineralization of 5 mM glycerol. The casamino acids stock solution was prepared at a concentration of 10 g/L and added to the media at a concentration of 494 mg O_2_/L as COD. Glucose and galactose stock solutions were prepared in deionized water at 1 M and autoclaved. The initial concentration of 2.5 mM glucose was measured as 414 mg O_2_/L as COD. The initial concentration of 2.5 mM galactose was measured as 426 mg O_2_/L as COD (Table S1). Palmitate and oleate stock solutions were made from their sodium salts at 1 M and autoclaved. The target initial concentration of 1 mM palmitate was measured as 264 mg O_2_/L as COD. The target initial concentration of 1 mM oleate was measured as 744 mg O_2_/L as COD (Table S1).

All high-density biomass samples were sterilized before use and added at total concentrations of 494 mg O_2_/L as COD. *Coccomyxa* was provided as oven-dried powder (31) and autoclaved at 121°C for 15 min (48). Duckweed was autoclaved at 121°C for 15 min. *Euglena* was provided pre-sterilized.

Biomass-laden filters collected from the deep layer of CM were used to inoculate the microcosms. Inside the anaerobic chamber (Coy Laboratory Products, Grass Lake, MI), filters were cut evenly into eight pie-shaped sections using sterile scissors (hereafter referred to as filter wedges). Four filters were required for this study, and the number of each filter was labeled in numerical order based on the order they were collected from the field. The correspondence between filters and microcosms is summarized in Table S2. These details are provided because it was shown in this research that each filter displayed subtle differences in microbial community composition and diversity. Additionally, two microcosms were set up using unsterilized *Coccomyxa* powder without a filter wedge to assess whether *Coccomyxa* contained any sulfate-reducing microbes.

Microcosms were prepared inside the anaerobic chamber in serum bottles amended with substrate and inoculated with a filter wedge. All stock solutions and high-density biomass were prepared and introduced to the corresponding microcosms within the anaerobic chamber. Stock solutions were purged with N_2_ for 10 min. Serum bottles were resealed with rubber stoppers and aluminum caps, purged with a 95:5% N_2_:CO_2_ gas mix for 20 min to approximate dissolved inorganic concentrations in CM, removed from the anaerobic chamber, and incubated on a shaker table at 18°C. Each treatment was prepared in quadruplicate.

### Analytical methods

Sulfide was measured using a modified Cline assay (49, 50). Briefly, 0.5 mL of suspension from each microcosm was mixed with 0.5 mL of 100 mM zinc acetate. Lamotte sulfide reagents A and B (LaMotte company, Chestertown, MD) were mixed in a ratio of 80–25 μL and then combined with the 1 mL suspension + zinc acetate for 40–60 min in the dark to digest sulfide solids. Cline-S(-II) was measured by absorbance at 670 nm using a standard curve. Sulfide standards were prepared by diluting a 1,000 ppm (31.25 µM) sodium sulfide standard solution (Aqua Solutions Inc., TX) to concentrations of 4, 1, 0.5, 0.25, and 0.125 µM under anaerobic conditions.

Glucose, galactose, and volatile fatty acids (VFAs) resulting from substrate oxidation were measured using a Shimadzu HPLC model LC20-AT equipped with a Bio-Rad HPX-87H column, a SIL 20-A autosampler, and two detectors: a Refractive Index (RI) detector (RID-20A), and a UV detector (SPD-M20A). The RI detector was used to measure glucose and galactose, and the UV detector was used to measure VFAs, including acetate, butyrate, and propionate. The oven temperature was set to 65°C, with 0.005 M sulfuric acid employed as the eluent, and the column retention time was 30 min. Prior to analysis, 1 mL of suspension was filtered (0.45 µm) and acidified (5 μL of 1 N H_2_SO_4_).

Dissolved metal(loid)s were measured using a Thermo Scientific iCAP 7400 ICP-AES and included Fe, Zn, Al, As, Na, Mn, Mg, and Si. Prior to analysis, 1 mL of suspension was filtered (0.45 µm) and acidified (1:1 [vol/vol] mixture of HCl and HNO₃).

pH was measured using a freshly calibrated Mettler Toledo LE422 SevenExcellence micro pH electrode immersed into a plastic vial of suspension.

### Statistical methods

All data are presented as mean ± standard deviation from n measurements. Due to the low number of replicates (*n* ≤ 4) and the large standard deviation of certain parameters within some treatments, non-parametric analysis of variance failed to detect differences among treatments. Therefore, the paired Mann-Whitney U test was used to assess statistical differences in chemical parameters between any two substrates (51). Statistical differences between the compared values were interpreted as being significant if *P* values were less than 0.05.

Kendall’s Tau correlation analysis was used to assess the relationships between the relative abundance of sulfate-reducing bacteria in the microcosms and the corresponding sulfide production rate and the maximum sulfide production.

### Kinetics calculation

The maximum zero-order sulfide production rate, d[S(-II)]/dt (equation 1), was used for kinetic comparisons between treatments. A zero-order rate law was used because it best fit the [S(-II)]-vs-time data and presented a simple kinetic comparison between treatments. The time period for the rate calculation started when sulfide production exceeded 0.05 mM/d and extended until production fell below 0.05 mM/d. Consequently, 2–4 time points were typically used to calculate these rates. The sulfide production rate was determined as follows:


(eq 1) $$Sulfide\;production\;rate\;(\text{μ}M/d)=\frac{S(\text{-}II)\;at\;the\;end\;point-S(\text{-}II)\;at\;the\;starting\;point}{end\;point\;time-starting\;point\;time}$$

### Thermodynamic calculations

Standard Gibbs free energy (ΔG_r_°) and Gibbs free energy of reaction (ΔG_r_’) on a per-carbon basis (kJ/mol C) were calculated for the oxidation of sugars, amino acids, and LCFAs coupled to sulfate reduction. Alanine and glycine were selected as representative amino acids. The calculations were based on the initial substrate concentrations, {CO₂(g)} = 0.05 atm, {SO₄²⁻} = 0.14 M, {NH₄^+^} = 0.01 M, pH = 4.2 and T = 291 K.

### DNA extraction and microbial community analysis

Biomass samples from microcosms for DNA extraction were collected during maximum sulfide production. Biomass was collected from 40 mL of suspension on Supor 200 Membrane Disc Filters (0.2 µm). DNA was also extracted directly from filter wedges used to inoculate the microcosms. DNA extraction was performed using the Qiagen DNeasy PowerWater Kit (Qiagen, Venlo, The Netherlands) following the manufacturer’s instructions. The concentration and quality of DNA extracts were measured by A260/A280 and A260/A230, respectively (Thermo Scientific NanoDrop One^C^). DNA extracts were frozen at −20°C until further use.

The V4 region of the 16S rRNA gene was amplified using 515F (5′-GTGYCAGCMGCCGCGGTAA-3′) and 806R (5′-GGACTACNVGGGTWTCTAAT-3′) primers. PCR reactions were set up using 2 µL of extracted DNA, 11.375 µL of sterile water, 10.625 µL of Ex Taq™ master mix with 806R reverse primer, and 1 µL of 515F forward barcoded primer (final concentration of 0.2 µM for each primer). The PCR thermocycler program included an initial denaturation step at 94°C for 3 min, followed by 35 cycles, each comprising 45 s at 94°C, 60 s at 50°C, and 90 s at 72°C. A final extension of amplicons was achieved by holding the reaction at 72°C for 10 min. PCR products were confirmed by analyzing the amplified products on a 2% agarose gel, with the presence of a band representing approximately 390 base pairs indicating successful amplification. The amplified region was sequenced on the Illumina MiSeq platform, employing 250 bp paired-end reads with a 500 bp insert by Mr. DNA (Shallowater, TX). Extracted DNA concentrations in each microcosm are listed in Table S3.

The 16S rRNA amplicon sequences were analyzed using DADA2. Raw sequences were assembled into contigs, and unique sequences were selected after filtration. Sequences were aligned with the Silva taxonomic training data (version 132) formatted for DADA2, facilitating the classification of amplicon sequence variants (ASVs) through sequence error models (52, 53). Relative abundances of ASVs in each microcosm were determined using rarefaction analysis at the genus level with a 0.03 cutoff. Highly abundant ASV sequences were matched against the NCBI BLAST database for species-level classification. For further analysis and visualization, output files from DADA2 and metadata files were employed in RStudio (version 4.2.2). Microbial community profiles were generated using the phyloseq package, and principal coordinate analysis (PCoA) was performed based on Bray-Curtis distances. The Shannon diversity profiles were also constructed using the phyloseq package (54), with data visualization enhanced by the ggplot2 package.

## RESULTS AND DISCUSSION

### Characteristics of high-density biomass

Analyses of high-density biomass showed substantially different compositions (Table 1). *Coccomyxa* had the highest protein content (48%), lowest starch content (1.1%), and second-lowest fiber content (0.2%). *Coccomyxa*’s measured protein content was consistent with a previous report (31). *Coccomyxa* was cultivated in a nutrient-rich culture medium, which explains its typically high protein content. *Euglena* was expected to have the highest carbohydrate content (e.g., roughly from 20% to 70%), which is primarily attributed to the presence of paramylon (β-1,3-glucan polysaccharide) in most *Euglena* species (55, 56); however, the measured starch content was 4.3%. *Euglena* had the lowest fiber content (0.1%) and the lowest S content (0.13%). Duckweed was found to contain the highest fiber content (17%), highest starch content (9.6%), and highest sulfur content (0.43%), along with a relatively low protein content (16%). These results are consistent with previous studies using this duckweed (Table 1). Note that these analyses represent only the specific materials tested in this study, and because of conditions related to harvesting and processing biomass, may not widely represent other natural forms of the biomass. All high-density biomass samples were processed in some manner before use in this study as described in the Materials and Methods section. Regardless of expected variations within each form of high-density biomass, our findings provide useful guidance for material selection for bioremediation practices.

**TABLE 1:** Compositional analysis from previous studies, proximate analysis, and elemental analysis of high-density biomass^*a*^

	*Coccomyxa*	*Euglena*	Duckweed
Compositional analysis from previous studies			
Carbohydrate %	25	66	15
Protein %	45	22	12
Lipid %	5.4	7.1	0.7
Reference	(31)	(55)	(39)
Proximate analysis			
Starch %	1.1	4.3	9.6
Protein %	48	15	16
Fat %	6.7	6.2	3.3
Fiber %	0.2	0.1	17
Elemental analysis of high-density biomass			
C %	50	43	34
N %	7.7	2.3	2.1
S %	0.40	0.13	0.43
P %	2.1	1.1	1.3
Fe (mg/kg)	2,190	35	2,410
Na (mg/kg)	217	950	8,340

^*a*^ All values are on a dry mass basis.

The N content in *Coccomyxa* (7.7%) was consistent with its high protein content (Table 1), which is typically accumulated in the biomass of numerous microalgal species grown under non-nutrient-limited conditions. The lower N contents in *Euglena* and duckweed (2.3% and 2.1%, respectively) were consistent with their lower protein contents (15% and 16%, respectively). The relatively high S content in *Coccomyxa* (0.4%) compared with common plant tissue (0.15%) (57) was likely due to its growth in a sulfate-rich environment. Additionally, *Coccomyxa*’s high Fe content and low Na content reflected its growing conditions, where Fe(III) was abundant in the nutrient-rich culture medium (up to 2 mM) (58) and Na less so in APLs.

### Utilization of high-density biomass

**Fig 1:**
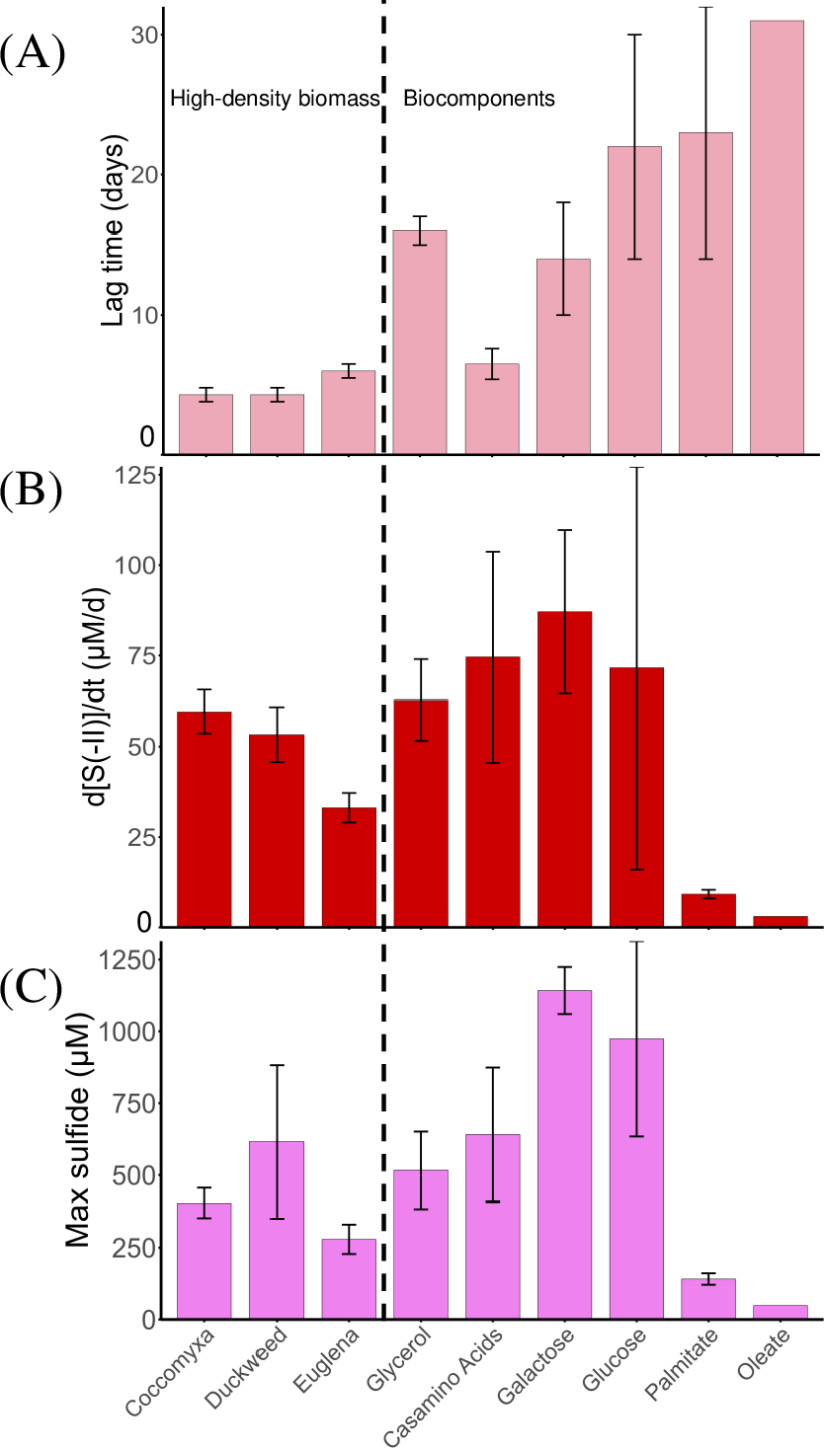
(**A**) Lag time, (**B**) zero-order sulfide production rate, and (**C**) maximum extent of sulfide production in microcosms amended with various substrates. Values are presented as mean ± standard deviation for N replicates. Mann–Whitney U tests were conducted for each parameter at a significance level of *P* < 0.05. Data for each microcosm are provided in Table S4.

All high-density biomass promoted dissimilatory sulfate reduction (Fig. 1; Table S4). With *Coccomyxa*, sulfide production began after a short lag time (4.3 ± 0.5 d), occurred at the fastest rate (59.6 ± 6.0 μM/d) (*P* < 0.05), and to a limited extent (404 ± 52 μM) (*P* < 0.05) for high-density substrates. With duckweed, sulfide production also began after a short lag time (4.3 ± 0.5 d), occurred at a fast rate (53.3 ± 7.6 μM/d), and resulted in the greatest extent of sulfide production (616 ± 266 μM) for high-density substrates. With *Euglena*, sulfide production began after a longer lag time (6.0 ± 0.5 d) and occurred at the slowest rate (33.1 ± 4.1 μM/d) (*P* < 0.05) and to the lowest extent (279 ± 51 μM) (*P* < 0.05) for high-density substrates. All high-density biomass-amended microcosms exhibited significantly shorter lag times than glycerol (*P* < 0.05), which has been recently used as a model substrate to promote the growth of SRB in APL (11). Sulfide production rate and maximum sulfide production in glycerol-amended microcosms were significantly higher than *Euglena*-amended microcosms (*P* < 0.05) but indistinguishable from *Coccomyxa*- or duckweed-amended microcosms. Lag times for sulfide production were significantly shorter with *Coccomyxa* and duckweed as compared to any of the single biocomponent-amended microcosms (*P* < 0.05). Shorter lag times in high-density biomass-amended microcosms may be due to its complex composition that includes multiple biocomponents—such as amino acids, sugars, vitamins, and other growth factors rather than a single compound. Among these biocomponents, the triacylglycerides composition of *Coccomyxa* and *Euglena* is abundant in saturated and monounsaturated fatty acids (59, 60), which are more efficiently oxidized by bacteria via the β-oxidation pathway. Additionally, unlike other substrates that primarily contain only C, H, and O, high-density biomass also provides essential macronutrients (e.g., N and P) and micronutrients. These nutrients likely facilitate microbial adaptation, similar to how yeast extract is commonly used to support microbial growth (61, 62). Specifically, *Coccomyxa sp*. has been found to accumulate vacuolar polyphosphate (polyP), which is suggested to serve as the vacuolar phosphorus storage form in chlorophyte algae (63) and can act as a phosphate source for microbial growth.

The increase in pH in the microcosms (highest with duckweed, lowest with *Euglena* [*P* < 0.05]) correlated directly to sulfide production, consistent with alkalinity production associated with greater sulfate reduction. As discussed below, all substrates (both high-density biomass and individual biocomponents) led to the enrichment of *Desulfosporosinus*, an acid-tolerant SRB genus (64). The increase in pH from 4.2 to values approaching pH 6 would create positive feedback to further stimulate the activity of neutrophilic SRB.

The maximum sulfide production from high-density biomass-amended microcosms was also converted to µM sulfide per mg L^−1^ COD (Table 2) to allow a more direct comparison of the utilization efficiency of biomass as an electron donor and assess the extent of unutilized reducing power. Duckweed exhibited a sulfide production efficiency of 1.25 ± 0.54 µM sulfide/mg L^−1^ COD, *Coccomyxa* 0.82 ± 0.10 µM sulfide/mg L^−1^ COD, and *Euglena* 0.56 ± 0.10 µM sulfide/mg L^−1^ COD. Among the high-density biomass tested in this study, duckweed remained the most efficient electron donor on a per-COD basis. However, these values are substantially lower than the theoretical maximum sulfide production of 15.6 µM sulfide/mg L^−1^ COD. The discrepancy was expected, given that a substantial portion of the high-density biomass remained visible in the microcosms, indicating incomplete degradation.

**TABLE 2:** Maximum sulfide production per unit of COD and final pH for microcosms amended with various substrates^*b*^

Substrate	*n* ^*a*^	Max sulfide (µM sulfide/mg-L COD)	Max pH
*Coccomyxa*	4	0.82 ± 0.10	5.81 ± 0.05
*Euglena*	4	0.56 ± 0.10	5.59 ± 0.09
Duckweed	4	1.25 ± 0.54	5.95 ± 0.06
Glycerol	4	1.04 ± 0.27	5.82 ± 0.02
Casamino acids	4	1.29 ± 0.47	6.12 ± 0.05
Glucose	3	2.35 ± 0.82	5.09 ± 0.46
Galactose	3	2.68 ± 0.19	4.85 ± 0.22
Palmitate	3	0.53 ± 0.08	5.55 ± 0.26
Oleate	1	0.06	5.73

^*a*^ Although all substrates were tested in quadruplicate, *n* = number of microcosms that produced sulfide.

^*b*^ Values presented as mean ± standard deviation for n replicates. Mann-Whitney U-test was conducted for each parameter at the level of significance *P* < 0.05. The data from each microcosm are listed in Table S4.

*Coccomyxa* is the predominant algae found in CM, and the *in situ* microbial community might already be adapted to utilize this substrate. Although *Euglena* is also an acid-tolerant algae, it stimulated sulfate reduction the least amongst high-density substrates. While duckweed is not an existing species in APLs, its high S content may have aided its promotion of sulfate reduction. Previous studies demonstrated that the addition of glycerol and elemental sulfur stimulated dissimilatory sulfate reduction more than glycerol alone in experiments very similar to the current study (11).

### Utilization of biocomponents

All biocomponents promoted dissimilatory sulfate reduction, although least effectively with lipids (Fig. 1; Fig. S2). With casamino acids, sulfide production required the shortest lag time (6.5 ± 1 d) (*P* < 0.05) and produced the highest final pH (6.12 ± 0.05) (*P* < 0.05) but showed no significant difference in sulfide production rate or maximum sulfide production. The shorter lag time was possibly attributed to the same factors described in the previous section, namely that the presence of macronutrients and micronutrients in amino acids, in addition to C, H, and O, facilitated microbial adaptation. Previous research on the bioremediation of APLs has demonstrated that supplementation with suitable nitrogen sources is essential for sustaining SRB activity. Nitrogen-containing organic substrates, such as amino acids and nitrogen-rich high-density organic matter (e.g., wood chips and sawdust), are effectively utilized by SRB in these systems, consistent with the shorter adaptation times observed for the amino acid– and high-density biomass–amended microcosms in this study. Therefore, in nitrogen-poor APLs such as CM, addition of a nitrogen-rich substrate (such as *Coccomyxa* vs *Euglena*) could better promote sulfate reduction. In other cases, nitrogen supplementation could be considered to enhance the effectiveness of bioremediation efforts in APLs (65).

With glucose and galactose, sulfide production required longer lag times (14 to 22 days) (*P* < 0.05), occurred at fast rates (71.6–87.1 μM/d), and resulted in the greatest maximum sulfide production (974–1,142 μM) among all substrates (*P* < 0.05). Glucose and galactose also exhibited the highest sulfide production per COD basis, with 2.35 ± 0.82 µM sulfide/mg L^−1^ COD for glucose and 2.68 ± 0.19 µM sulfide/mg L^−1^ COD for galactose, whereas casamino acids yielded 1.30 ± 0.47 µM sulfide/mg L^−1^ COD (Table 2). These results suggest that monosaccharides, particularly glucose and galactose, which are abundant in microalga polysaccharides (reducing power source for biomass-degrading bacteria), are the most efficiently utilized electron donors on a per-COD basis (*P* < 0.05), likely because the carbon atoms in sugar monomers are more readily biochemically oxidized compared with oxidation than those in amino acids (66, 67). The rates of sulfide production in monosaccharide-amended microcosms were indistinguishable compared with casamino acid-amended microcosms (74.6 ± 29.1 μM/d) and high-density biomass (*P* > 0.05), except for *Euglena*. With lipids, sulfide production required the longest lag times (23–31 days), occurred at the slowest rates (3.0–9.2 μM/d), and resulted in the lowest extent of sulfide production (49–141 μM or 0.07–0.53 µM sulfide/mg L^−1^ COD) among all substrates (*P* < 0.05). With oleate, only one of the replicate microcosms produced sulfide. Subtle differences in microbial community composition and diversity between filters and filter wedges from the same filter may have contributed to variable results between replicates prepared with oleate. Since palmitate and oleate have relatively high pK_a_ values (9.85 for oleate and 8.60 for palmitate) (68), LCFAs predominantly exist in their unprotonated forms under our experimental conditions (pH ~ 4.2). This unprotonated form is more readily able to cross cell membranes, leading to cellular damage (69). Low solubilities of lipids could have made them more challenging for microbial oxidation (70). All these factors might contribute to the inefficient utilization of LCFAs in the current study.

Free energy calculations could explain the differences in lag times (R^2^ = 0.511) but did not directly correlate with the sulfide production rate (R^2^ = 0.052) (Fig. 1 and Table 3; Fig. S3). Alanine and glycine (reactions R1 and R2) were chosen as representative amino acids for these calculations, and their oxidation, coupled with sulfate reduction, was the most thermodynamically favorable on a per carbon basis. Amino acid-microcosms displayed a shorter lag time before sulfide production began, compared with monosaccharide-amended microcosms. Glycerol has an energy yield comparable with that of monosaccharides on a per-carbon basis; correspondingly, they exhibited a similar lag time. Monosaccharides (reactions R3 and R4) yield less energy than amino acids on a per-carbon basis; correspondingly, they exhibited longer lag times. However, they were utilized at faster rates and to a greater extent of sulfide production. LCFAs yield the lowest energy per carbon (reactions R5 and R6), which may further contribute to the longer lag times and lower rate for the utilization of LCFAs.

**TABLE 3:** Standard Gibbs free energy (ΔG_r_^0^) and Gibbs free energy of reaction (ΔG_r_') (kJ/mol C) calculated for reactions carried out by microorganisms in these experiments^*a*^

Rxn#		ΔG_r_^0^ (298 K)	ΔG_r_' (291 K)	
R1: Alanine	C_2_H_5_NO_2_ + 0.75SO_4_^2-^ + 2.5H^+^ == 2CO_2_ + 0.75H_2_S + NH_4_^+^ + H_2_O	−167	−153	kJ/mol C
R2: Glycine	C_3_H_7_NO_2_ +1.5SO_4_^2-^ + 4H^+^ == 3CO_2_ + 1.5H_2_S + 2NH_4_^+^ + 2H_2_O	−171	−165	kJ/mol C
R3: Glucose	C_6_H_12_O_6_ + 3SO_4_^2-^ + 6H^+^ == 3H_2_S + 6CO_2_ + 6H_2_O	−121	−118	kJ/mol C
R4: Galactose	C_6_H_12_O_6_+ 3SO_4_^2-^ + 6H^+^ == 3H_2_S + 6CO_2_ + 6H_2_O	−141	−137	kJ/mol C
R5: Palmitate	C_18_H_33_O_2_^-^ + 12.75SO_4_^2-^ + 26.5H^+^ == 12.75H_2_S + 17H_2_O + 18CO_2_	−106	−97.1	kJ/mol C
R6: Oleate	C_16_H_31_O_2_^-^ + 11.5SO_4_^2-^ + 24H^+^ == 11.5H_2_S + 16 H_2_O + 16CO_2_	−97.3	−88.4	kJ/mol C
R7: Glycerol	C_3_H_8_O_3_ + 1.75SO_4_^2-^ + 3.5H^+^ == 1.75H_2_S + 3CO_2_ + 4H_2_O	−146	−136	kJ/mol C

^*a*^ ΔG_r_' refers to pH = 4.2; [Palmitate] = [Oleate] = 1*10^-3^ M; [Glucose] = [Galactose] = 2.5*10^-3^ M, [Alanine] = [Glycine] = 2.5*10^-3 ^M, [Glycerol] = 5*10^-3^ M, {CO_2_(g)}= 0.05 atm, {SO_4_^2-^}= 0.14 M, [NH_4_^+^] = 0.01 M, {H_2_S(aq)} = 10^-6^ M, T=18°C = 291 K.

### Metal(loid) removal

**Fig 2:**
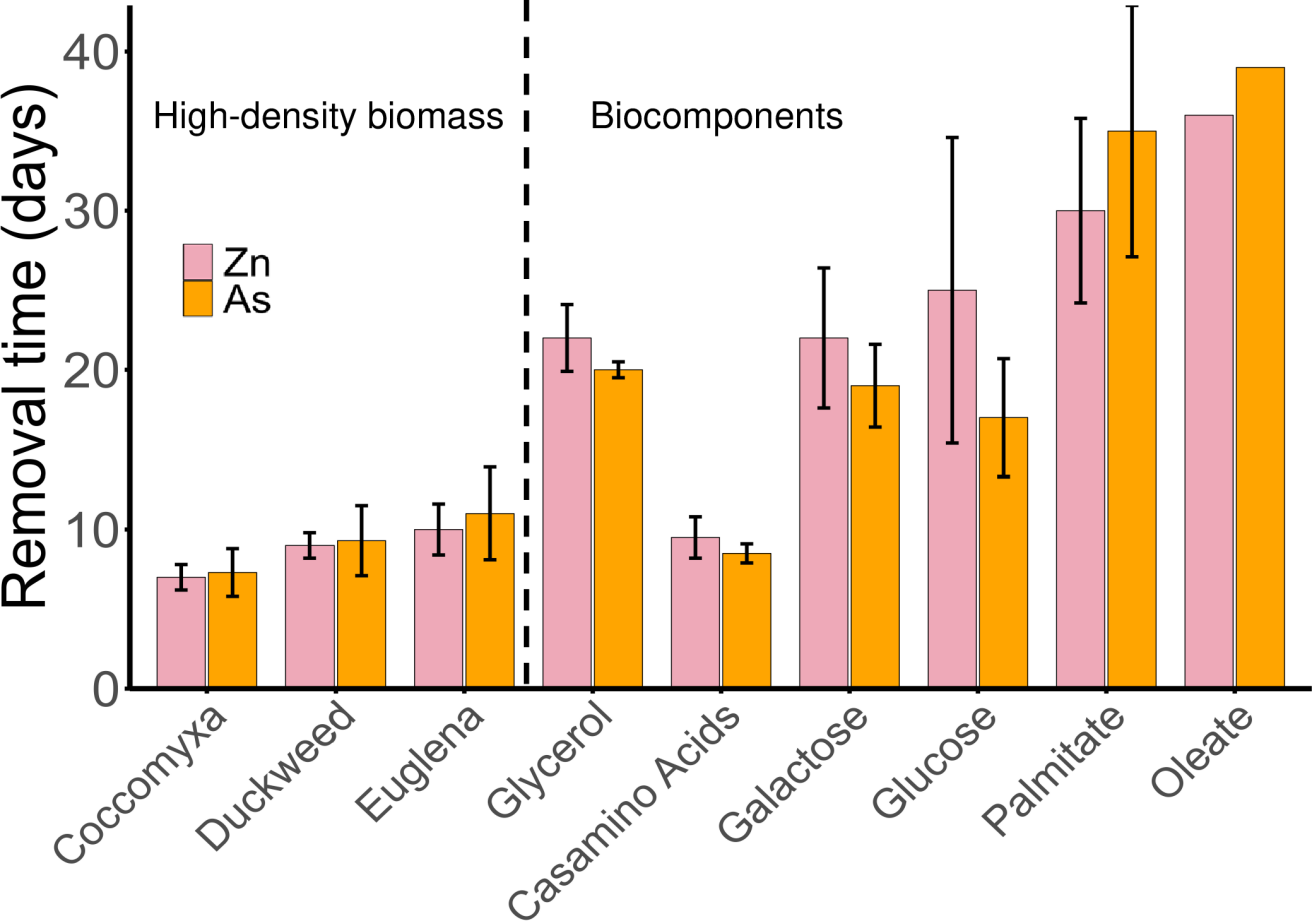
Times required for complete removal of arsenic and zinc with different substrates. Values presented as mean ± standard deviation for N replicates. Mann-Whitney U-test was conducted for each parameter. Data from each microcosm are listed in Table S4. Oleate-amended microcosms only have one replicate that produced sulfide.

As expected, metal(loid) removal occurred coincident with sulfide production. All microcosms produced enough sulfide to completely immobilize Zn and As from solution. Zn and As were targeted because they are the two major metal(loid)s in the deep layer of CM that are prone to precipitation by sulfide (21). Microcosms amended with high-density biomass required the shortest time for complete removal of Zn and As (*P* < 0.05) (Fig. 2), consistent with their short lag times for sulfide production (Fig. 1). All high-density biomass-amended microcosms also showed faster Zn and As removal than glycerol. Biocomponent-amended microcosms that required a longer lag time for sulfide production (i.e., monosaccharides and lipids) also required longer times for removal of Zn and As. Metal(loid)s were removed faster in casamino acid-amended microcosms compared with those amended with other biocomponents and glycerol (*P* < 0.05). Compared with Zn and As, Fe removal (data not shown) was a less sensitive indicator of metal removal due to its higher initial concentration (12 mM Fe vs 0.2 mM Zn vs 0.006 mM As) and the higher solubility of FeS compared with ZnS (21, 71).

Arsenic removal times were faster than Zn removal in experiments with glycerol, casamino acids, glucose, and galactose, which could be due to faster precipitation kinetics of realgar (As_2_S_3_) compared with sphalerite/wurtzite (ZnS), which is consistent with *in-situ* observations in CM (21). However, As removal is not significantly faster than Zn removal in experiments using high-density biomass. According to Table 2, glucose- and galactose-amended microcosms reached a final pH of approximately 5, whereas the high-density biomass-amended microcosms reached a final pH of nearly 6. Therefore, the higher pH may have somehow limited As₂S₃ precipitation compared with ZnS precipitation.

### Accumulation of volatile fatty acids (VFAs) during substrate utilization

**Fig 3:**
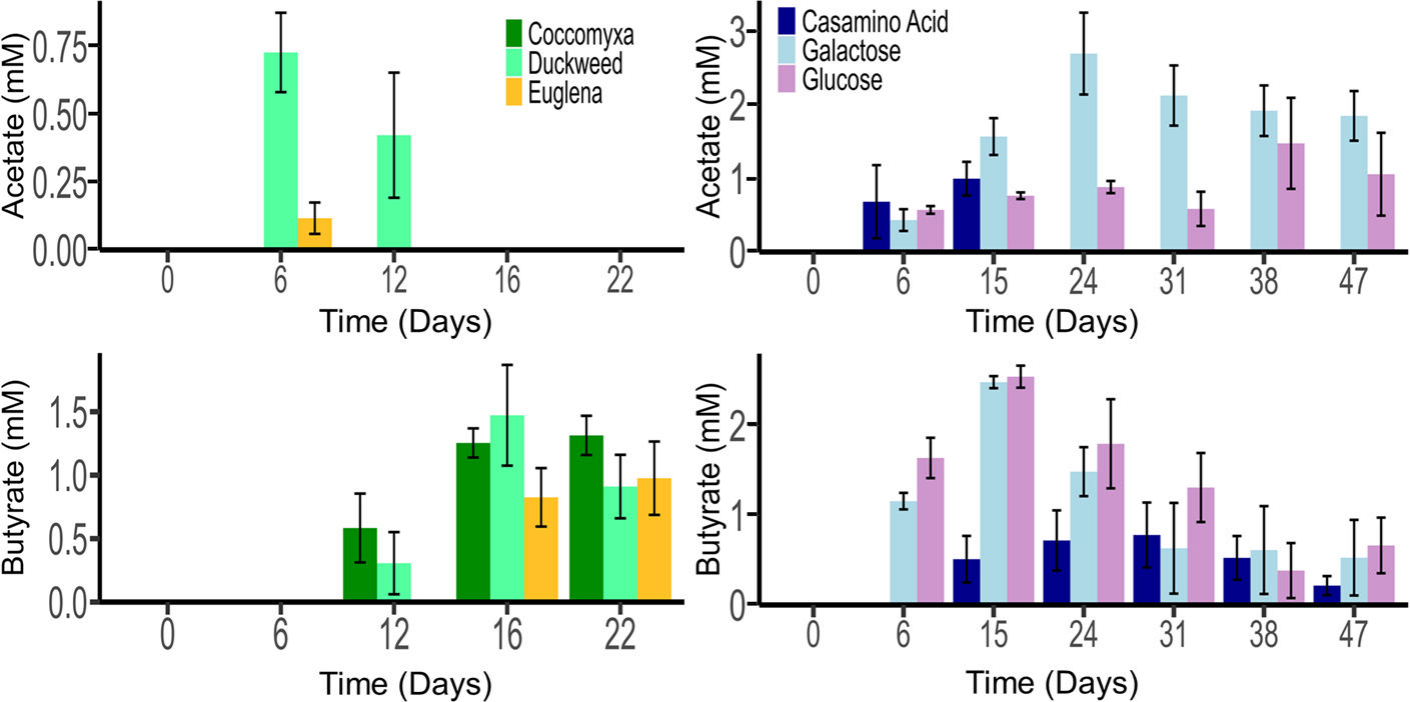
Acetate and butyrate over time in microcosms amended with high-density biomass (left) and biocomponents (right). Microcosms amended with lipids are not shown because they produced no VFAs. No glucose or galactose remained after 15 days.

Acetate and butyrate were produced from the oxidation of all high-density biomass and from casamino acids and monosaccharides (Fig. 3). No VFAs were detected from LCFAs, and no other VFAs were produced from any of the substrates. In *Euglena* and duckweed-amended microcosms, acetate was detected early in the incubation period but was subsequently consumed. In all high-density biomass-amended microcosms, butyrate was detected towards the middle of the incubation period and remained until the end of the incubation period (22 d).

The pattern of early acetate production, followed by acetate consumption and butyrate production, was also observed in casamino acid-amended microcosms. In comparison, monosaccharide-amended microcosms produced both acetate and butyrate throughout the incubation period, with higher concentrations of acetate at the end of the incubation period (47 days). No VFAs were detected in LCFA-amended microcosms, possibly because hydrogen was produced through β-oxidation and subsequently utilized as an electron donor for SRB or because VFAs were generated via β-oxidation but were rapidly consumed by SRB, preventing their accumulation from being captured in our measurements (72, 73). Greater accumulation of VFAs in monosaccharide-amended microcosms was consistent with their higher maximum sulfide concentrations compared with casamino acid-amended microcosms (Fig. 3). Acetate (pK_a_ = 4.76) is toxic to cells under low-pH conditions because it remains largely in its unprotonated form (74–76), and *Desulfosporosinus* is unable to utilize acetate as an electron donor (64). Therefore, since the monosaccharide-amended microcosms still maintained low pH at the end of incubation, acetate consumption was suppressed until conditions improved. The accumulation of more acetate, compared with butyrate, may partially explain the lower final pH values measured in monosaccharide-amended microcosms (pH 4.85–5.09), compared with casamino acids (pH 6.12) (Table 2). Since ~1.8 mM acetate was accumulated in galactose-amended microcosms, whereas ~0.2 mM butyrate was accumulated in casamino acid-amended microcosms, and acetate (pK_a_ = 4.76) is a stronger acid than butyrate (pK_a_ = 4.82), the discrepancy can result in the former having a 0.5-unit lower pH than the latter, as predicted by the Henderson-Hasselbalch equation (equation 2).


(eq. 2) $$pH={pK}_{a}+\log\frac{\left[conjugate\;base\right]}{\left[weak\;acid\right]}$$

Additionally, the higher acetate concentrations in monosaccharide-amended microcosms provided greater buffer capacity to the solution (equation 3).


(eq. 3) $$\beta =2.303\left[\frac{k_{w}}{\left[H^{+}\right]}+[H^{+}]+\frac{[H^{+}]\;C\cdot K_{a}}{{\left([H^{+}]+K_{a}\right)}^{2}}\right]$$

where β represents the buffer capacity, defined as the amount of base required to change the pH of a buffer solution by 1 unit, and *C* denotes the total concentration of weak acids—acetate or butyrate in this study. As a result, these microcosms were more resistant to pH increases caused by alkalinity production by dissimilatory sulfate reduction, leading to a lower final pH.

The temporal patterns of VFA production from high-density biomass closely resemble those observed for casamino acids, with both exhibiting an initial phase of acetate accumulation, followed by butyrate accumulation. These results suggest that amino acids in the high-density biomass were likely the preferential substrates for sulfate reduction. Therefore, high-density biomass with higher protein content (and higher N content) may be more effective for a remediation approach based on biosulfidogenesis, as amino acids serve as protein monomers, and *D. acididurans* possesses a complete set of genes for protein degradation into individual amino acids (77).

### Changes in microbial communities under different amendments

**Fig 4:**
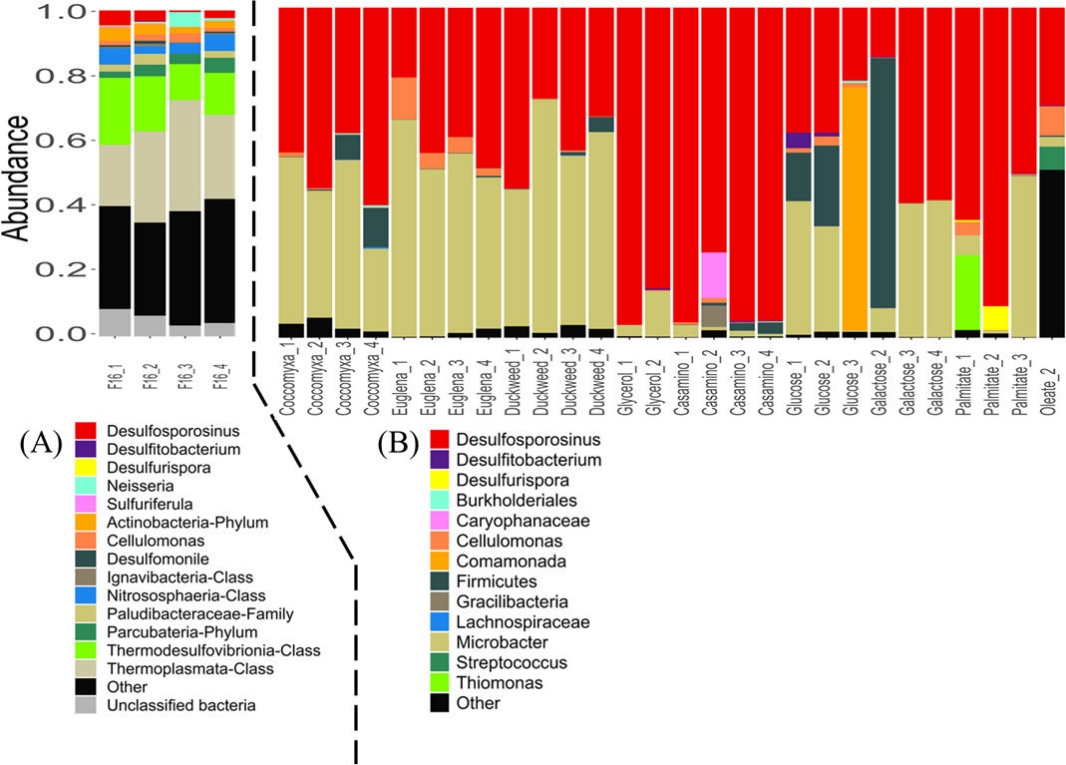
(**A**) Microbial communities recovered from filter wedges collected from the deep layer of CM (adapted from reference 11). F16_1 to F16_4 represent four replicates of microbial communities directly extracted from filter No. 16, which was collected from the field. (**B**) Relative abundance of prokaryotes based on 16S rRNA gene amplicons. Each column is labeled with the corresponding microcosm name and replicate number (e.g. Coccomyxa_1 represents the replicate 1 of microcosms supplemented with *Coccomyxa* as the substrate. Only the 13 most abundant taxa are shown; all remaining taxa are grouped under “Other.”

All amendments enriched for microbial communities that were significantly different from the *in-situ* community collected from the deep layer of CM (Fig. 4). The *in-situ* community was developed under oligotrophic conditions and included low relative abundances of SRB. In particular, the SRB genus *Desulfosporosinus* ranged from 0.3% to 4.6% (9, 11) relative abundance in the *in-situ* community yet was greatly enriched with all substrates tested in this study. Using NCBI BLAST, this taxon was found to have a 100% V4 region sequence match to *Desulfosporosinus acididurans* (64). This bacterium is highly opportunistic and has been shown to proliferate much faster than other SRB, such as *Desulfurispora*, *Desulfovibrio,* and *Desulfomonile,* under organic substrate-amended conditions in an incubation column study using deep-layer water collected from an APL located near CM (11, 78).

In *Coccomyxa-*amended microcosms, the relative abundance of *Desulfosporosinus* ranged from 39% to 59%. In other microcosms amended with high-density biomass, the relative abundance of *Desulfosporosinus* ranged from 21% to 49% in the *Euglena*-amended treatments and from 27% to 55% in the duckweed-amended treatments.

Despite variations in the relative abundance of *Desulfosporosinus* among replicates, any correlation between SRB abundance and sulfide production was weak (Kendall’s τ, *P* > 0.05). For instance, in *Euglena*-amended microcosms, regardless of the relative abundance of *Desulfosporosinus* (21% to 49%), all microcosms produced 257–368 µM sulfide (Table S4). Similarly, duckweed-amended microcosms produced 379–928 µM sulfide, whereas the relative abundances of *Desulfosporosinus* ranged from 27% to 55%. Finally, in *Coccomyxa*-amended microcosms, where the relative abundance of *Desulfosporosinus* ranged from 39% to 59%, all microcosms produced 357–477 µM sulfide. Similarly, the relative abundance of *Desulfosporosinus* showed a weak correlation with sulfide production rates (Kendall’s τ, *P* > 0.05). Details of the sulfide production parameters for each replicate are provided in Table S4.

*Desulfosporosinus* was also enriched in biocomponent-amended microcosms. In particular, the relative abundance of *Desulfosporosinus* ranged from 75% to 95% in casamino acid-amended microcosms. Considering the similarity of VFA accumulation in temporal patterns during the incubation with high-density biomass (Fig. 3), this result suggests that amino acids are good substrates for the enrichment of *Desulfosporosinus. Desulfosporosinus* was enriched to lesser extents in microcosms amended with glucose (22% to 38%), galactose (15% to 59%), palmitate (50% to 90%), and oleate (30%) also suggesting a preference for amino acids utilization by *Desulfosporosinus*.

In biocomponent-amended microcosms, the rate and extent of sulfide production (Fig. 1) was not directly dependent on the relative abundance of *Desulfosporosinus* (Kendall’s τ, *P* > 0.05). For example, the greatest rates and extent of sulfide production occurred in monosaccharide-amended microcosms, systems with lower amounts of *Desulfosporosinus* (Fig. 4). As another example, the greatest relative abundance of *Desulfosporosinus* occurred in casamino acid-amended microcosms, systems that produced less sulfide compared with monosaccharides.

*Microbacter* was the second most abundant taxa in most of the microcosms and even the most abundant in a few. Using NCBI BLAST, this *Microbacter* species was identified as *Microbacter margulisiae* isolated from sediment in the Tinto River, Spain (79). The abundance of *Microbacter* was associated with the abundance of *Desulfosporosinus*, consistent with previous studies that have shown that *M. margulisiae* is significantly enriched alongside SRB under acidic sulfate-reducing conditions (79–81). *Microbacter* is not identified as an SRB but is capable of fermentation, producing acetate, lactate, and propionate as major fermentation products. Therefore, *Microbacter* was less abundant in casamino acid- and glycerol-amended microcosms, where these substrates could be directly respired rather than fermented (44, 79). In comparison, *M. margulisiae* was more abundant in the high-density biomass–amended microcosms, where organic substrates were predominantly present in complex forms. Therefore, fermentation of these complex organics into simpler compounds was likely necessary before they could be utilized by SRB (82, 83). In addition, nitrogen (e.g., as NH_4_^+^) produced from fermentation of complex nitrogen-containing organic substrates could be utilized by SRB to enhance their growth (65). *M. margulisiae* is often found in co-occurrence with SRB in APLs (84–86).

*Cellulomonas* is a genus of cellulose degraders, and it was present (> 1%) in several microcosms where the relative abundance of *Desulfosporosinus* was low (e.g., with *Euglena*, glucose, and lipids). *Burkholderiales*, identified as a minor taxon in (9), was detected in certain microcosms. Its presence is likely related to its abundance in the original inoculum, which may vary across different filters.

*Desulfurispora* is an SRB genus that was detected only in palmitate-amended microcosms, with relative abundances ranging from approximately 0.3%–8.4%. Few studies have described *Desulfurispora* to date (87, 88), and its characteristics remain largely unknown. Given that *Desulfurispora* was also slightly enriched in the microcosm supplemented solely with elemental sulfur in our previous study (11), where the sulfide production rate was extremely slow, it may possess greater adaptability under slower sulfide-producing conditions. The SRB genus *Desulfitobacterium* was detected in microcosms amended with galactose (0.15%) and glucose (0.8%–3.4%). Since *Desulfitobacterium* cannot utilize glucose or galactose as electron donors (89–91), we suspect that it utilized intermediate products of sugar respiration or fermentation, such as citrate, pyruvate, and other related compounds (92). Other species listed in Fig. 4B will be summarized and discussed in Table S5.

Two major taxa recovered from the inoculum collected from the deep layer of CM, uncultured *Thermoplasmatales* and *Thermodesulfovibrio*, were not detected (no amplicon) in any microcosm. *Thermoplasmatales* is an acidophilic and thermophilic taxon at family level that is frequently found in APLs (7, 93, 94). The closest genome related to the *Thermodesulfovibrio* species *in situ* was *Thermodesulfovibrio yellowstonii* (9). *T. yellowstonii* is a strict anaerobic SRB with a temperature range of 40°C–70°C and an optimal pH between 6.8 and 7.0 (95). To date, all known *Thermodesulfovibrio* isolates are neutrophiles or alkaliphiles (96, 97). These taxa may inhabit less acidic sediments in CM but could not tolerate the low pH and low temperature of the lab incubations.

**Fig 5:**
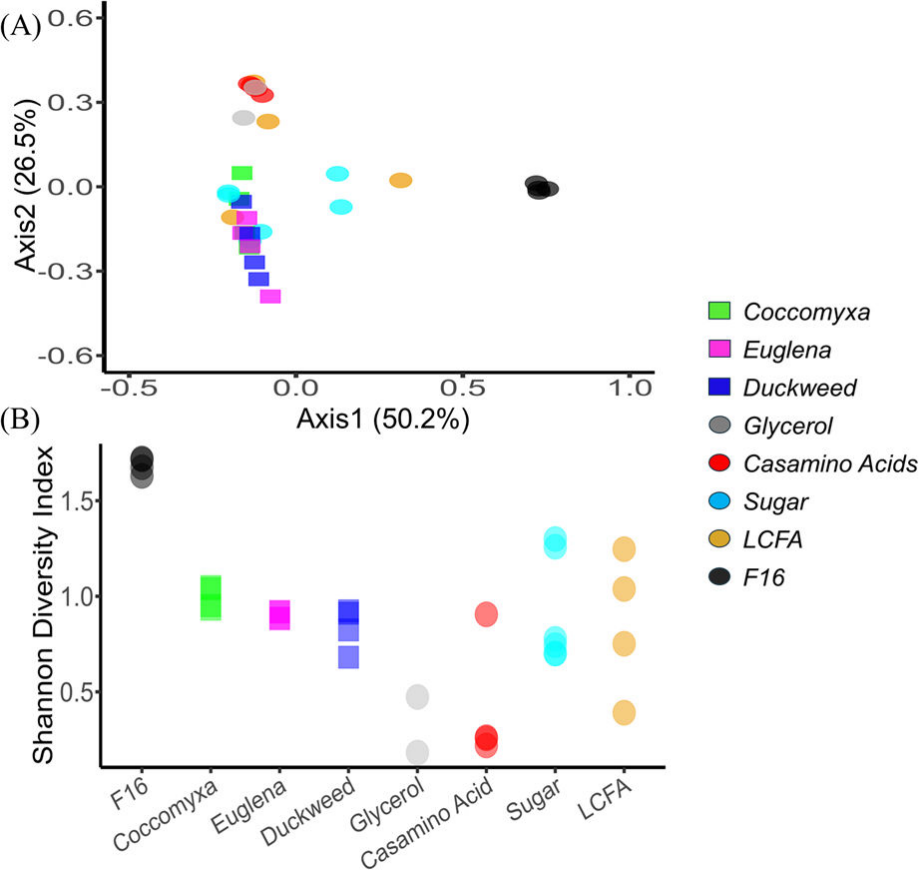
(**A**) Principal coordinate analysis (PCoA) results based on Bray-Curtis pairwise dissimilarity. (**B**) Shannon diversity indices of ASVs (16S rRNA sequence counts) in microcosms and the microbial community on the filter wedge (F16). High-density biomass is depicted as squares, and biocomponents are represented by circles.

Based on Bray-Curtis pairwise dissimilarity and Shannon diversity (both using V4 region of 16S rRNA gene), all enriched microbial communities were distinct from the *in-situ* community used as inoculum (Fig. 5A), and all were less diverse (Fig. 5B). The loss of diversity was expected considering an excess of electron donor introduced selective pressure to enrich for those microbes capable of utilizing the added substrate (98). Moreover, maintaining strictly anaerobic conditions in the laboratory introduced additional selective pressure, inhibiting the growth of facultative microbes that may exist *in situ* (9, 10, 26). The PCoA of the Bray-Curtis metric showed clear groupings for the glycerol and the casamino acid–amended microcosms, which were located close to each other, indicating similar microbial community structures. In contrast, the high-density biomass–amended microcosms (squares in Fig. 5) clustered tightly together but were positioned apart from the other groups, reflecting distinct community compositions primarily driven by the greater relative abundance of *Microbacter*.

Microbial communities enriched by the various amendments did not correlate to the rate and extent of sulfide production, whereas parameters related to sulfide production were statistically different with different amendments (Fig. 1 and Table 2). The data suggests that the substrates had a greater impact in stimulating dissimilatory sulfate reduction compared with the enriched microbial community.

### Engineering applications

The primary remediation goal for the deep layer of a permanently stratified APL like CM would be to remove major harmful metal(loid)s from the dissolved phase so that they cannot be transported to the surrounding environment. A series of conceptual calculations is presented here to show how this could occur in CM. Based on representative geochemical conditions in the deep layer of CM, ~1.67 mM Zn and ~0.23 mM As would have to be removed as sulfide minerals. Zn would presumably be removed as ZnS, thus requiring 1.67 mM of S(-II) to be produced for its removal. Arsenic would presumably form As_2_S_3_, the predominant As sulfide found in the deep layer of CM (99), thus requiring 0.35 mM of S(-II) to be produced (= 3/2*0.23 mM). Therefore, a total of ~2 mM S(-II) would have to be produced to remove As and Zn, initially neglecting the formation of FeS.

The amount of high-density biomass needed to produce ~2 mM S(-II) can be estimated based on these experimental results (Fig. 1). For example, the addition of 0.38 g/L of *Coccomyxa* produced 0.40 mM S(-II), resulting in a sulfide production ratio of 1.06 mM S(-II)/g *Coccomyxa*. Using the same calculation method yields sulfide production ratios of 0.57 mM S(-II)/g *Euglena* and 1.04 mM S(-II)/g duckweed (Table S6). Although high-density biomass materials were added to the microcosms at the same concentrations based on COD, the different extents of substrate oxidation (i.e., measured in terms of sulfate reduction) reflect different biochemical oxygen demand (BOD) concentrations of these materials. Differences between COD and BOD reflect that a portion of the high-density biomass materials may not be labile enough for biological utilization under sulfate-reducing conditions.

It should be noted that a critical constraint for bioremediation would be to maintain permanent stratification of the lake. Permanent stratification of an APL is due to the high density caused by elevated salinity in the deep layer of CM; substrate addition would have to be carefully monitored to avoid removing too many dissolved species from the deep layer. Lake turnover must be avoided, as oxygen intrusion from the surface layer could reverse the remediation effects by disrupting biosulfidogenesis (16), negating its bioremediation benefits. Additionally, lake turnover would negatively impact the ecosystem of APLs. For instance, obligate anaerobes residing in the deep layer would likely perish if transported to the oxygenated surface layer (100, 101).

Therefore, enough metal(loid) ions must remain in the deep layer during bioremediation to prevent vertical mixing. Timely monitoring of ion concentrations and density is critical during remediation to avoid significant overdosing. Fortunately, the stratification of CM is quite stable, given that approximately 120 mM of Fe(II) and 126 mM SO₄²⁻ remain in the deep layer of CM, being much higher than the iron and sulfate contents of the chemocline (19 mM Fe(II) and 41 mM SO₄²⁻) (20). As a result, the density of the deep layer of CM (ρ = 1,015 mg/cm^3^) is significantly higher than that of the upper layer (ρ = 1,001 mg/cm^3^) and chemocline (ρ = 1,002 mg/cm^3^) (102), so that a lake turnover due to density homogenization during remediation would be highly unlikely. Furthermore, metallic sulfides settled into the sediment of the APLs would not be severely affected by lake turnover and primarily protected from reoxidation (20). In any case, timely monitoring would be recommended to ensure the high-density biomass is not buried in the sediment before it is utilized in the water column, as these particles do not resuspend into the water column in such a deep, permanently stratified lake (103), although their utilization or dissolution could lead to upward diffusion of electron donors into the deep water column.

In a hypothetical case for the remediation of CM, we propose setting the target S(-II) production at 3 mM by the end of a defined remediation period as a safety factor to ensure complete removal of the major harmful metal(loid)s in the deep layer of CM, Zn and As, while maintaining sufficient Fe(II) in the deep layer, assuming that the rate of metal(loid) removal (which typically requires a few months) is much faster than the rate at which new dissolved metal(loid)s accumulate in the deep layer of CM. Further investigation and more comprehensive studies are needed to establish a reasonable remediation goal and safety factor, and to develop engineering strategies to prevent the settling of high-density biomass into the sediment.

The total mass of high-density biomass required for addition to the deep layer of CM was calculated based on an estimated volume of approximately 112,800 m³, derived from the bathymetry of CM (8). Consequently, approximately 350 US tons of dried *Coccomyxa* or pelletized duckweed would need to be introduced into the deep layer to achieve remediation goal (i.e., 3 mM S(-II) production) (Table S6), which corresponds to approximately 2.81 metric tons per million liters (10.6 US tons per Mgallon) of water treated. Biomass production in the surface layer and chemocline is insufficient to provide the amount of biomass required for deep-layer remediation (8, 27); therefore, stimulating growth through nutrient supplementation or adding external biomass may be necessary (104). In comparison, based on default design specifications for passive treatment systems in the United States (105, 106), a sulfate-reducing bioreactor designed to treat 3,785 L per min of acid mine drainage (AMD) would require approximately 9,520 metric tons of biomass (e.g., a combination of hay, manure, and wood chips). Assuming this biomass sustains sulfate reduction for 10 years, the system would treat approximately 19,900 million liters of AMD, yielding a biomass requirement of 0.48 metric tons per million liters (2.01 US tons per Mgallon). Therefore, the remediation efficiency of using high-density biomass to treat the deep layer of CM is comparable with that of similar biomass-based strategies applied to AMD.

Moreover, using high-density biomass offers several advantages. *Coccomyxa* can be directly harvested from the surface layer and chemocline of CM. Duckweed can be collected from nature and requires only minimal processing in laboratory facilities before use. Furthermore, we found that unsterilized *Coccomyxa* collected from CM could trigger biosulfidogenesis with a comparable lag time (6.8 ± 1.0 days) and sulfide production rates (56.8 ± 1.5 µM/day) to those observed with sterilized *Coccomyxa* (Fig. 1). This is likely because *Coccomyxa* harbored SRB native to CM, which were enriched during incubation. Therefore, autoclaving the high-density biomass for substrate-native organisms may not be necessary. Further investigation is warranted to assess whether bioremediation efficiencies differ significantly between sterilized and unsterilized high-density biomass. If no substantial difference is observed, autoclaving costs could be reduced. In addition, we conducted a lab test to determine the sinking velocity of pelletized high-density biomass. The duckweed pellets (6 mm diameter, 2.5 cm long) sank at a rate of ~15 cm/s in lake water. *Coccomyxa*, in the form of small pellets, sank at a rate of 2 cm/s. These data suggest that pelletized high-density biomass can sink to the bottom of CM (40 m) much faster (e.g., ~3–30 min) than the lag period for SRB to utilize them (~days, as discussed in Fig. 1 above). Therefore, they can reach the remediation area without significant loss during transport from the surface to the deep part. In comparison, soluble organic substrates such as glycerol, glucose, and galactose must be added directly to the deep layer, which requires infrastructural effort and leads to substantial additional costs.

### Conclusions

In this study, we tested a new potential approach to remediate the deep layer of a permanently stratified APL by utilizing high-density biomass to facilitate dissimilatory sulfate reduction, thus immobilizing harmful metal(loid)s by forming metal(loid)-sulfide precipitates. The acid-tolerant SRB *Desulfosporosinus* was enriched and served as the major facilitator for dissimilatory sulfate reduction for this process, but its relative abundance was weakly correlated to sulfide production efficiency. High-density biomass serves as a favorable substrate for the acid-tolerant SRB *Desulfosporosinus* inhabiting the deep layer of CM. High-density biomass was utilized more rapidly than simple organic substrates such as glycerol, glucose, and galactose. Besides, they can naturally sink, avoiding the infrastructural cost for delivering soluble or floating substrates to the deep layer. The “indirect delivery” of *Coccomyxa* enables internal bioremediation by cultivating it in the surface layer and allowing for passive transport to the deep layer. The “direct delivery” of external pelletized high-density biomass, such as duckweed, has proven successful in the laboratory setup, indicating a potential success of this strategy to be applied in practical remediation. Utilization of amino acids experienced a shorter adaptation period among the biological monomers examined in this study, whereas monosaccharides led to a greater sulfide production rate. Thus, we infer that amino acid-rich (and possibly S-rich) biomass may undergo a shorter adaptation period, whereas sugar-rich biomass might result in a higher sulfide production rate during remediation. To our knowledge, this is the first published work to propose the application of high-density biomass for bioremediation in APLs in the Iberian Pyrite Belt.

## Data Availability

Data were deposited at the National Center for Biotechnology Information (NCBI) and can be found under the BioProject accession number PRJNA1347290.

## SUPPLEMENTAL MATERIAL

Supplemental material aem.02369-25-s0001.docx Figures S1 to S3; Tables S1 to S6.

ASM does not own the copyrights to Supplemental Material that may be linked to, or accessed through, an article. The authors have granted ASM a non-exclusive, world-wide license to publish the Supplemental Material files. Please contact the corresponding author directly for reuse.
