# Supplementary material for: High-density biomass as a substrate for stimulating biosulfidogenesis in the deep layer of stratified acidic pit lakes
Source: Appl Environ Microbiol. 2026 Jan 12;92(2):e02369-25. doi: 10.1128/aem.02369-25 (PMC12915300; doi:10.1128/aem.02369-25)
Supplement: Supplemental material — Figures S1 to S3; Tables S1 to S6. [file aem.02369-25-s0001.docx]

**Appendix**


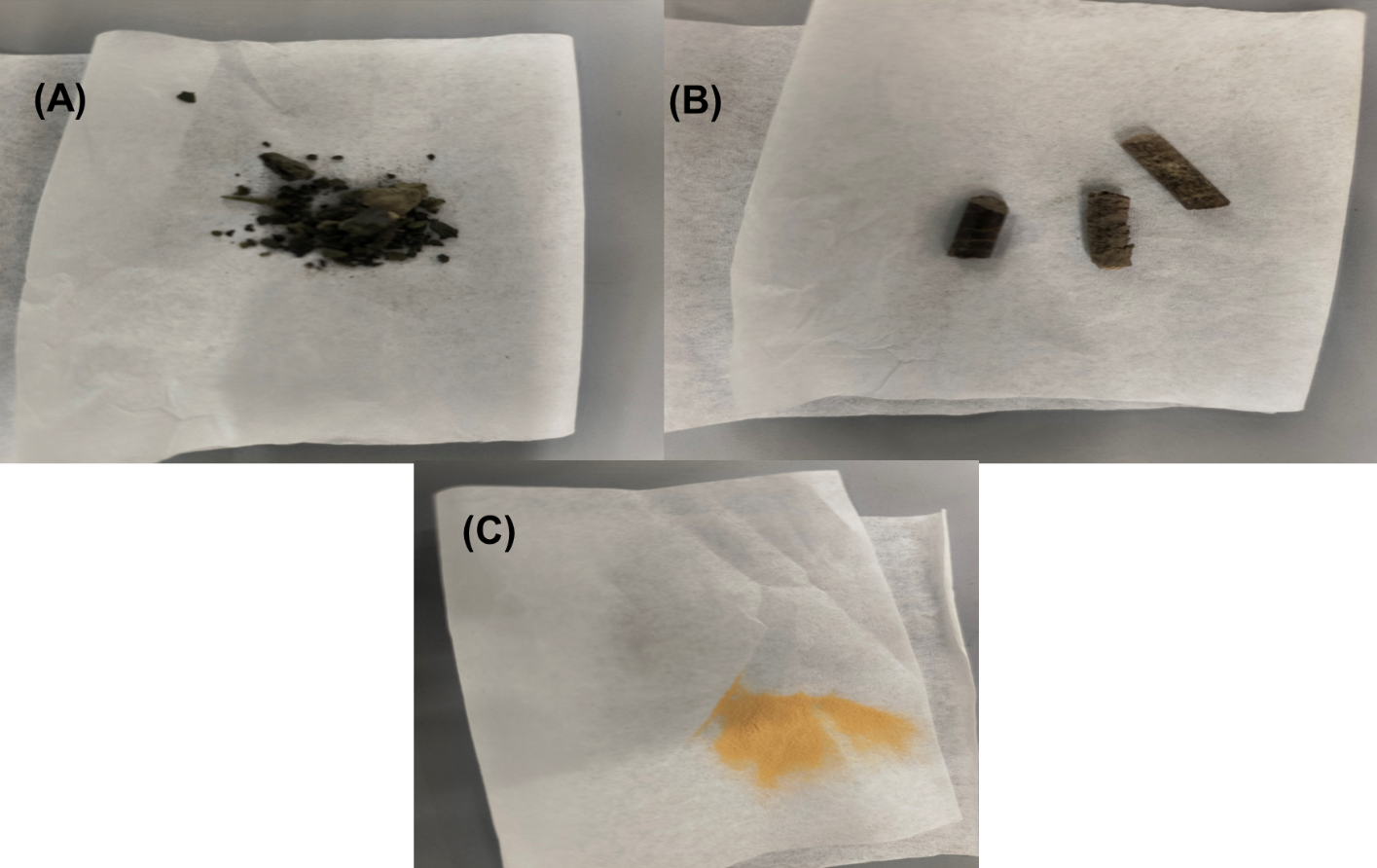


**Figure S1**. Visual appearance of the high-density biomass used in this experiment: (A) *Coccomyxa onubensis* in fine pelletized form, (B) Duckweed pellets (~1.5-2 cm long, 6-mm in diameter), and (C) *Euglena* in fine powder form.


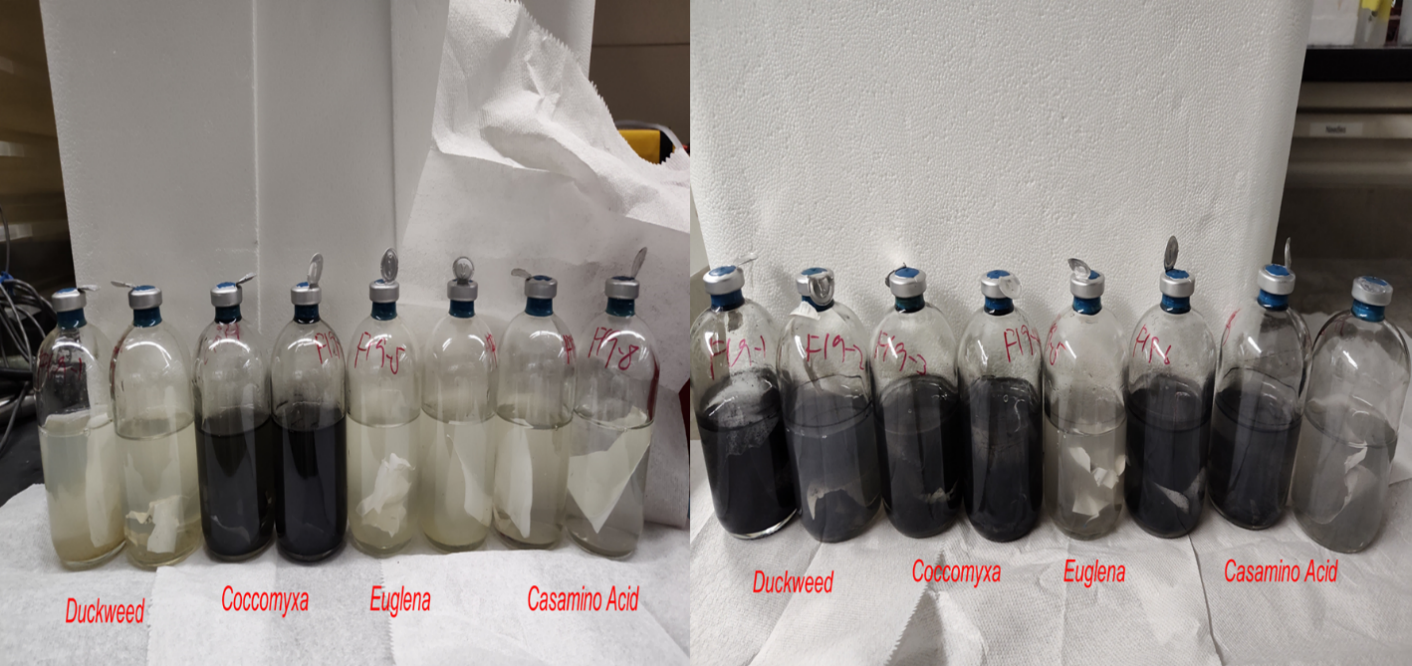


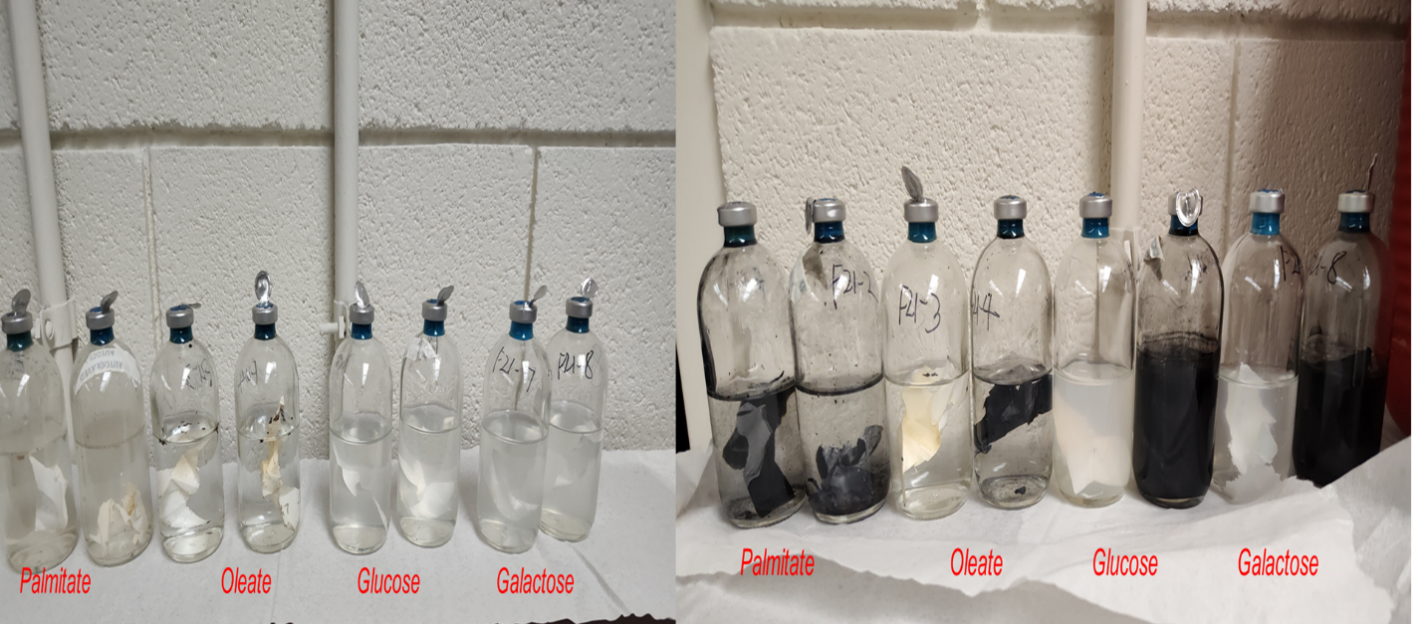


**Figure S2**. Photographs of the microcosms supplied by biocomponents and casamino acids at (Top) (A) Day 9 (B) Day 15. Microcosms supplied by *Coccomyxa* both turned black at day 9, indicating a more sulfide accumulation in these microcosms at this timepoint, while essentially all other microcosms also turned turbid at this timepoint, which is attributed to the formation of ZnS before Fe-S minerals. (Bottom) (C) Day 15 (D) Day 31. On day 15, both replicates of microcosms amended with glucose and galactose turned turbid, suggesting the formation of ZnS. By day 31, both replicates amended with palmitate, one replicate each amended with oleate, glucose, and galactose, turned black, with the color being notably darker in the microcosms amended with glucose and galactose. In the case of the microcosms amended with palmitate, only the filter wedge turned black while the liquid phase remained clear. The remaining replicate of the glucose- and galactose-amended microcosms stayed turbid, indicating sulfide formation sufficient to precipitate Zn but not enough to initiate FeS formation, which would result in a color change.


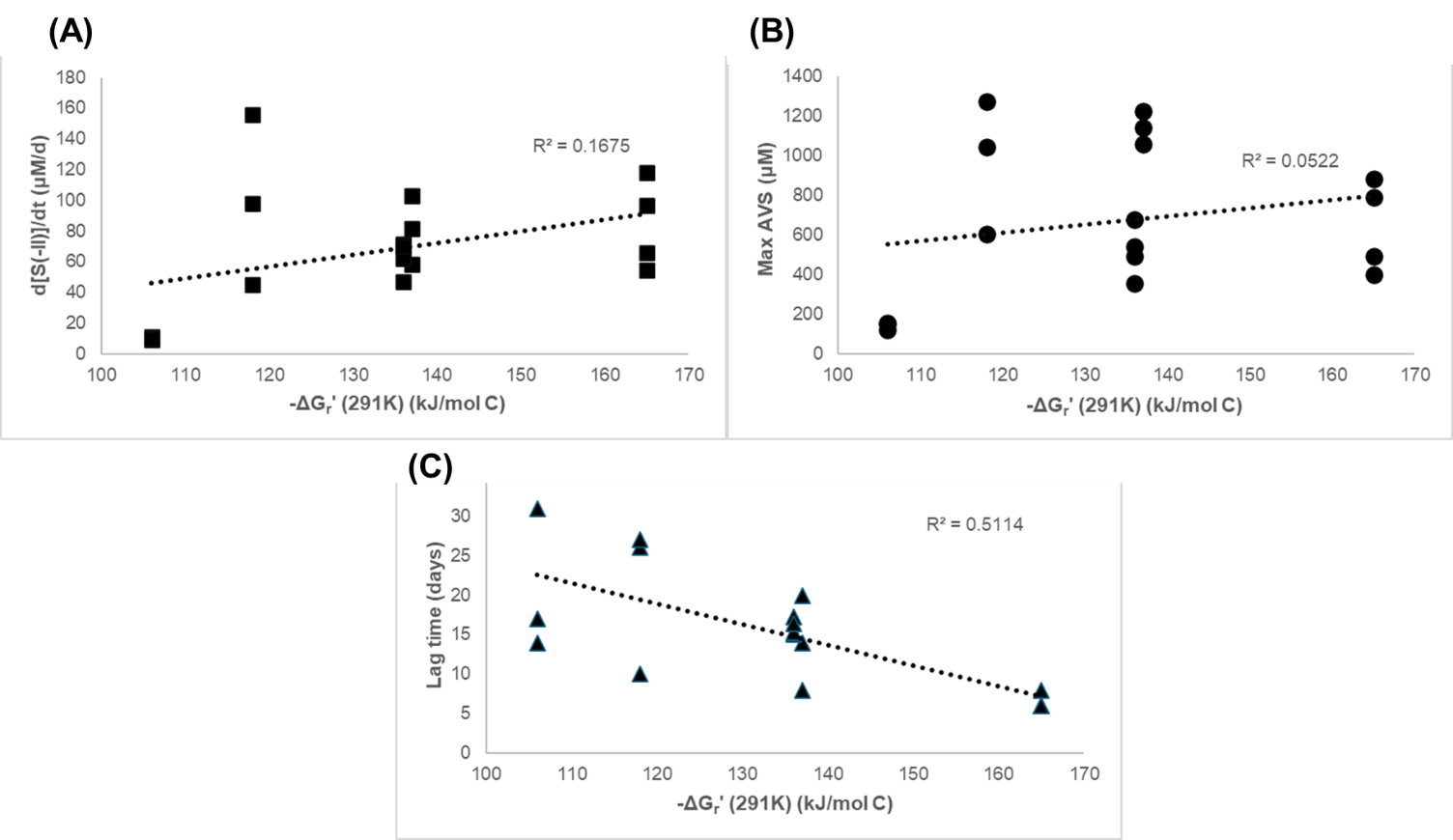


**Figure S3**: Multi-panel figure showing the correlation between -ΔGr' at 291K and (A) d[S(-II)]/dt (B) Max AVS and (C) Lag time within biocomponents and glycerol amended microcosms. R-square values are presented in the figures.

**Table S1.** Top: Concentrations of solid-phase carbon substrates and casamino acids added equivalent to the COD demand of 5 mM glycerol (494 mg COD/L), along with the corresponding calculated carbon concentrations (mM) based on their respective carbon percentages. Bottom: COD equivalents and carbon concentrations (mM) for polysaccharides (glucose and galactose) and lipids (palmitate and oleate) added to the medium at known concentrations.

| Target COD: 494 mg/L = 5 mM glycerol | | | |
| --- | --- | --- | --- |
| **Substrates** | **Mass concentrations added (mg/L)** | **COD (mg/L)** | **C Concentration (mM C)** |
| *Coccomyxa* | 403 | 494 | 16.6 |
| *Euglena* | 522 | 494 | 18.9 |
| Duckweed | 605 | 494 | 25.0 |
| Casamino Acids | 618 | 494 | 25.8 |
|  | **Concentrations added (mM)** | **COD (mg/L)** | **C Concentration (mM C)** |
| Glucose | 2.5 | 414 | 15.0 |
| Galactose | 2.5 | 426 | 15.0 |
| Palmitate | 1 | 264 | 16.0 |
| Oleate | 1 | 744 | 18.0 |

**Table S2**. The correspondence between filter numbers and the inoculated microcosms

| Filter No. | Microcosms |
| --- | --- |
| 21 | Glucose_1 and 2  Galactose_1 and 2  Palmitate_1 and 2  Oleate_1 and 2 |
| 17 | Glucose_3 and 4  Galactose_3 and 4  Palmitate_3 and 4  Oleate_3 and 4 |
| 19 | Coccomyxa_1 and 2  Euglena_1 and 2  Duckweed_1 and 2  Casamino acids_1 and 2 |
| 20 | Coccomyxa_3 and 4  Euglena_3 and 4  Duckweed_3 and 4  Casamino acids_3 and 4 |

**Table S3**. DNA concentrations in every individual microcosm

| **Microcosm** | **DNA concentration (ng/uL)** |
| --- | --- |
| *Coccomyxa*_1 | 41.6 |
| *Coccomyxa*_2 | 225.3 |
| *Coccomyxa*_3 | 94.1 |
| *Coccomyxa*_4 | 36.6 |
| *Euglena*_1 | 14.1 |
| *Euglena*_2 | 11.4 |
| *Euglena*_3 | 29.0 |
| *Euglena*_4 | 22.2 |
| Duckweed_1 | 18.8 |
| Duckweed_2 | 17.5 |
| Duckweed_3 | 29.3 |
| Duckweed_4 | 62.7 |
| Casamino Acids_1 | 15.4 |
| Casamino Acids _2 | 6.8 |
| Casamino Acids _3 | 29.3 |
| Casamino Acids _4 | 34.2 |
| Glucose_1 | 24.8 |
| Glucose_2 | 48.5 |
| Glucose_3 | 13.6 |
| Galactose_2 | 41.5 |
| Galactose_3 | 22.2 |
| Galactose_4 | 13.1 |
| Palmitate_1 | 8.6 |
| Palmitate_2 | 7.4 |
| Palmitate_3 | 37.5 |
| Oleate_2 | 1.8 |

**Table S4**. Chemical parameters in Table 2 and Table 4 in every individual microcosm.

| **Microcosm** | **Lag time (days)** | **d[S(-II)]/dt (μM/d)** | **Max AVS (μM)** | **Max pH** | **[Zn] removal (days)** | **[As] removal (days)** |
| --- | --- | --- | --- | --- | --- | --- |
| *Coccomyxa*_1 | 5 | 64.6 | 357 | 5.84 | 8 | 6 |
| *Coccomyxa*_2 | 4 | 63.8 | 383 | 5.82 | 7 | 6 |
| *Coccomyxa*_3 | 4 | 54.0 | 371 | 5.73 | 7 | 9 |
| *Coccomyxa*_4 | 6 | 67.8 | 477 | 5.83 | 6 | 8 |
| *Euglena*_1 | 6 | 36.6 | 257 | 5.64 | 8 | 11 |
| *Euglena* _2 | 6 | 41.7 | 275 | 5.45 | 12 | 7 |
| *Euglena* _3 | 6 | 32.7 | 268 | 5.65 | 10 | 10 |
| *Euglena* _4 | 7 | 33.2 | 368 | 5.61 | 10 | 14 |
| Duckweed_1 | 4 | 51.8 | 379 | 5.99 | 8 | 6 |
| Duckweed _2 | 4 | 58.4 | 748 | 5.86 | 10 | 11 |
| Duckweed _3 | 4 | 50.5 | 928 | 5.96 | 9 | 10 |
| Duckweed _4 | 5 | 40.1 | 411 | 5.98 | 9 | 10 |
| Casamino Acids_1 | 6 | 54.8 | 397 | 6.19 | 10 | 9 |
| Casamino Acids_2 | 8 | 65.8 | 791 | 6.16 | 11 | 9 |
| Casamino Acids_3 | 6 | 118 | 884 | 6.07 | 8 | 8 |
| Casamino Acids_4 | 6 | 96.9 | 493 | 6.17 | 9 | 8 |
| Glucose_1 | 26 | 97.9 | 1275 | 4.97 | 35 | 23 |
| Glucose_2 | 10 | 156 | 1043 | 5.52 | 16 | 15 |
| Glucose_3 | 27 | 44.9 | 605 | 4.61 | 29 | 18 |
| Galactose_2 | 8 | 81.4 | 1060 | 5.02 | 14 | 17 |
| Galactose_3 | 14 | 58.2 | 1224 | 4.75 | 17 | 16 |
| Galactose_4 | 20 | 103 | 1142 | 4.58 | 25 | 21 |
| Palmitate_1 | 14 | 9.04 | 118 | 5.79 | 26 | 26 |
| Palmitate_2 | 17 | 11.4 | 148 | 5.73 | 26 | 29 |
| Palmitate_3 | 31 | 9.93 | 157 | 5.55 | 36 | 41 |
| Oleate_2 | 31 | 3.04 | 48.7 | 5.73 | 36 | 39 |

**Table S5.** Summary of other microbial species detected in the microcosms and their potential function

| **Taxa** | **Functions** | **Reference** |
| --- | --- | --- |
| *Firmicutes* | Phylum, possibly unclassified SRB, as most other taxa within *Firmicutes* in this research were SRB. |  |
| *Comamonada* | Family in *Burkholderiales,* possibly involved in the nitrogen cycle and sulfur metabolism. | Lünsmann et al.,, 2015; Manos 2022 |
| *Gracilibacteria* | Candidatus phylum, found in anaerobic environments, exhibiting a host-dependent lifestyle. | Fujii et al.,, 2022; Figueroa et al.,, 2024 |
| *Lachnospiraceae* | Strict anaerobes, specializing in the fermentation of complex biocomponents, particularly polysaccharides. | Boutard and Cerisy 2014; Jiang et al.,, 2024 |
| *Streptococcus* | A genus in the phylum *Firmicutes*, known as a human pathogen. | Patterson 1996;  Spellerberg 2015 |
| *Thiomonas* | A genus in *Comamonada*, likely sharing similar functions within the community. | Moreira and Amils 1997 |
| *Caryophaneceae* | Found in marine ecosystems, involved in the metabolism of aromatic compounds and heavy metals. | Gupta and Patel, 2020 |

**Table S6.** Required amounts of selected high-density biomass to achieve 3 mM sulfide production in the deep layer of CM, with a total volume of 112,800 m³.

|  | Concentration of substrates (g/L) | Sulfide Production (mM) | Sulfide production per unit of substrates (mM/g-L) | Substrates required (metric tons) |
| --- | --- | --- | --- | --- |
| *Coccomyxa* | 0.38 | 0.404 | 1.06 | 319 |
| *Euglena* | 0.49 | 0.279 | 0.57 | 594 |
| Duckweed | 0.59 | 0.616 | 1.04 | 325 |
